# SUN *vs* BEV*þ*IFN in first-line mRCC therapy: no evidence for a statistically significant difference in progression-free survival

**DOI:** 10.1038/sj.bjc.6605446

**Published:** 2010-01-05

**Authors:** M Nuijten, G Mickisch

**Affiliations:** 1 Erasmus University Rotterdam, Health Economic Department, PO Box 1738, Rotterdam 3000 DR, The Netherlands; 2 Centre of Operative Urology, Robert-Koch-Str. 34a, Bremen 28277, Germany

**Sir,**

It is with great interest that I read the article by Thompson Coon *et al*, as I agree with the authors that there is a need for performing indirect treatment comparisons in order to offer guidance on therapy decisions to oncologists.

As this is an important topic, I would like to outline some methodological issues that are related to the Thompson Coon *et al* analysis.

## *P*-value calculation approach

Using a one-sided *t*-test to calculate a *P*-value, as performed by Thompson Coon *et al* (2009), has to be regarded as a questionable approach, as two-sided *t*-tests are the common approach in clinical research and should be expected in such an analysis by the clinical readers of *BJC*. Even if this unusual approach is explicitly described, the findings in the abstract are misleading, as they seem to show statistical proof on a significant difference comparing sunitinib (SUN) with bevacizumab (BEV)+interferon alpha-2a (IFN) in first-line metastatic renal cell carcinoma. To elaborate, Thompson Coon *et al* (2009) simply calculated the *P*-value as the proportion of Bayesian Markov Chain Monte-Carlo (MCNC) trials in which the hazard ratio (HR) for any comparison exceeded the indirect comparison HR of ‘1’. In contrast, the authors should have reported the number of instances in which the lower confidence limit of HR for each iteration exceeded 1 or simply tested the hypothesis that Log(HR)=0, using the standard *z*-statistic. If a valid statistical method is used (e.g., that from Snedecor and Cochran, 1989), this would have led to a *P*-value that indicates a non-significant difference between SUN and BEV+IFN. This non-significant difference between both therapy options using a two-sided *t*-test is obvious, as the indirect comparison HR of SUN *vs* BEV+IFN exceeded ‘1’ in the upper 95% confidence interval limit.

## MCMC sampling

The authors performed an indirect comparison using Bayesian MCMC sampling, with IFN as a common comparator, adopting a fixed-effect model. Point estimates and 95% confidence intervals were calculated from 100 000 simulated draws from the posterior distribution after a burn-in of 10 000 iterations.

This up-sampling of point estimates has most likely led to more narrow confidence intervals that may be responsible for possible significant differences between treatments. Taking into account the fact that the SUN pivotal trial included 750 patients (Motzer *et al*, 2007) and the pooled BEV trials included 1381 patients (Escudier *et al*, 2007; Rini *et al*, 2008), the chosen number of simulation samples overpowers that of base trials. In general, it is recommended to either select the number of iterations on the total number of patients or the expected number of patients in the country of interest; hence, around 2000 simulation samples would have been an appropriate choice.

## Indirect comparison approach

Although the authors name the Bucher *et al* methodology (Bucher *et al*, 1997) as ‘a fairly simple analytical approach’, the Canadian Agency for Drugs and Technologies in Health (CADTH) (Wells *et al*, 2009a) and others (Tudur *et al*, 2002; Song *et al*, 2003) have identified this method as the most suitable approach for performing indirect treatment comparisons. The Bucher *et al* methodology is to be regarded as the gold-standard method for indirect treatment comparisons, as it is transparent and excludes the risk of producing misleading results that may be produced by up-sampling the power of base trials.

## Base data used

An adequate indirect comparison approach should consider pivotal trials performed under similar conditions as comparable, and assume highest data quality (independent radiology review of PFS, blinded as opposed to the open-label study).

If the author pooled a pivotal trial (Escudier *et al*, 2007) and an investigator-initiated trial (Rini *et al*, 2008) for BEV+IFN, the same approach should have been applied for SUN using the pivotal trial (Motzer *et al*, 2007) and first-line outcomes from the expanded access study (Gore *et al*, 2007).

## Own findings

According to our own ongoing research that uses the gold-standard Bucher *et al* methodology and an independent review PFS HR of pivotal trials (SUN *vs* IFN 0.538; 95% CI: 0.439–0.658 (Motzer *et al*, 2007), BEV+IFN *vs* IFN 0.571 (0.450–0.723) (Escudier *et al*, 2009)), there is no statistically significant evidence for a difference in efficacy with respect to PFS between SUN and BEV+IFN-α. The indirect comparison HR was 0.942 (95% CI 0.69–1.29; two-sided *t*-test *P*=0.71).

CADTH offers an open-access tool (Wells *et al*, 2009b) that enables researchers worldwide to re-perform and validate our findings.

As indirect treatment comparisons are increasingly used in medical decision making, I considered it important to draw attention to methodological issues for the benefit of those both performing and using the results of these comparisons.
