# Letter to the Editor: SUN *vs* BEV*þ*IFN in first-line mRCC therapy: no evidence for a statistically significant difference in progression-free survival

**DOI:** 10.1038/sj.bjc.6605613

**Published:** 2010-03-16

**Authors:** M Nuijten, G Mickisch

**Correction to:** *British Journal of Cancer* (2010) **102**, 232–233. doi: 10.1038/sj.bjc.6605446

Upon publication of this Letter to the Editor, it was noted that the affiliation for Dr Mark Nuijten had been presented incorrectly. The correct affiliation for Dr Nuijten is ‘Ars Accessus Medica in Amsterdam, Dorpsstraat 75 1546 LG, The Netherlands’.

